# Glutamate neurons are intermixed with midbrain dopamine neurons in nonhuman primates and humans

**DOI:** 10.1038/srep30615

**Published:** 2016-08-01

**Authors:** David H. Root, Hui-Ling Wang, Bing Liu, David J. Barker, László Mód, Péter Szocsics, Afonso C. Silva, Zsófia Maglóczky, Marisela Morales

**Affiliations:** 1 Neuronal Networks Section, Integrative Neuroscience Research Branch, National Institute on Drug Abuse, 251 Bayview Blvd Suite 200, Baltimore, MD 21224, USA; 2 Department of Psychology, Szent Borbála Hospital, H-2800, Tatabánya, Hungary; 3 Laboratory of Cerebral Cortex Research, Institute of Experimental Medicine of the Hungarian Academy of Sciences, H-1083, Budapest, Hungary; 4 Cerebral Microcirculation Section, Laboratory of Functional and Molecular Imaging, National Institute of Neurological Disorders and Stroke, 49 Convent Drive Bldg 49 Room 3A72, Bethesda, MD 20892-4478, USA.

## Abstract

The rodent ventral tegmental area (VTA) and substantia nigra pars compacta (SNC) contain dopamine neurons intermixed with glutamate neurons (expressing vesicular glutamate transporter 2; VGluT2), which play roles in reward and aversion. However, identifying the neuronal compositions of the VTA and SNC in higher mammals has remained challenging. Here, we revealed VGluT2 neurons within the VTA and SNC of nonhuman primates and humans by simultaneous detection of VGluT2 mRNA and tyrosine hydroxylase (TH; for identification of dopamine neurons). We found that several VTA subdivisions share similar cellular compositions in nonhuman primates and humans; their rostral linear nuclei have a high prevalence of VGluT2 neurons lacking TH; their paranigral and parabrachial pigmented nuclei have mostly TH neurons, and their parabrachial pigmented nuclei have dual VGluT2-TH neurons. Within nonhuman primates and humans SNC, the vast majority of neurons are TH neurons but VGluT2 neurons were detected in the pars lateralis subdivision. The demonstration that midbrain dopamine neurons are intermixed with glutamate or glutamate-dopamine neurons from rodents to humans offers new opportunities for translational studies towards analyzing the roles that each of these neurons play in human behavior and in midbrain-associated illnesses such as addiction, depression, schizophrenia, and Parkinson’s disease.

The presence of dopamine neurons in the nonhuman primate and human ventral tegmental area (VTA) and substantia nigra pars compacta (SNC) has been documented for nearly half a century^1^,^2^. Nonhuman primate studies have shown that VTA and SNC dopamine neurons play fundamental roles in decision making, motivation, reward, aversion, and learning^3^,^4^. Clinical findings have led to the hypothesis that VTA and SNC midbrain dopamine neurons are involved in a variety of disorders, including addiction^5^, schizophrenia^6^, depression and other mood disorders^7^, and movement disorders^8^.

As in nonhuman primates and humans, dopamine neurons are highly concentrated in the mouse and the rat VTA and SNC, and also play fundamental roles in decision making, motivation, reward, aversion, and learning^9^,^10^. The multiple functions of the midbrain dopamine neurons are likely to be mediated by diverse phenotypes of dopamine neurons and by their associated neuronal networks, some of which have local origins. Regarding the rodent VTA cellular composition, in addition to dopamine neurons (identified by expression of tyrosine hydroxylase; TH), the rodent VTA has GABA neurons (identified by expression of glutamic acid decarboxylase or vesicular GABA transporter), and glutamate neurons (identified by expression of vesicular glutamate transporter 2; VGluT2)^11^. In addition to these three major phenotypes of VTA neurons, the rodent VTA has subsets of neurons that exhibit combinatorial neurotransmitter-releasing characteristics. Some of these combinatorial neurons are glutamate-dopamine neurons and others are glutamate-GABA neurons^12^,^13^,^14^,^15^,^16^.

In addition to anatomical studies showing that the rodent VTA has a diverse cellular composition, the participation of diverse VTA neurons in motivated behavior has been addressed by application of optogenetic approaches in transgenic rodents. Findings from these optogenetic studies have revealed discrete midbrain neuronal phenotypes that appear to play distinct roles in reward, aversion, motivation, or learning^17^,^18^,^19^,^20^,^21^,^22^,^23^,^24^,^25^,^26^,^27^. In contrast to the advances made in identifying unique neuronal phenotypes and their functions in the mouse and rat midbrain, the cellular composition of the dopaminergic midbrain in higher mammals, such as the nonhuman primate and human, is unknown. The lack of information on the cellular composition of the human midbrain contributes to a translational gap in our understanding of the neurobiology of addiction, depression, and other midbrain-associated disorders. To start addressing this gap, here we combined *in situ* hybridization and immunohistochemistry to determine the possible presence of glutamate or glutamate-dopamine neurons within the VTA and SNC in nonhuman primates and humans.

## Results

To determine whether glutamate neurons are present within the VTA or SNC of nonhuman primates and humans, we combined immunodetection of TH (to establish the boundaries and subdivisions of the VTA and SNC), with radioactive *in situ* hybridization to identify the neuronal expression of transcripts encoding VGluT2 mRNA (as antibodies against VGluT2 do not label cell bodies of VTA-VGluT2 neurons)^11^.

### Detection and distribution of VGluT2-only neurons, TH-only neurons and dual VGluT2-TH neurons within the subdivisions of the marmoset VTA and SNC

In a pilot study, we found neurons expressing VGluT2-mRNA intermingled with TH-neurons in the VTA and SNC of both marmoset (*Callithrix jacchus*) and squirrel (*Saimiri sciureus*) monkeys. However, due to limited access to primate brain tissue, the quantitative cellular analysis of VGluT2-expressing neurons and TH-expressing neurons was confined to the marmoset midbrain. For each subdivision of the VTA or the SNC, we counted all identified neurons expressing VGluT2-mRNA (VGluT2-neurons), TH immunoreactivity (TH-neurons) or a combination of both (VGluT2-TH neurons).

**Figure 1:**
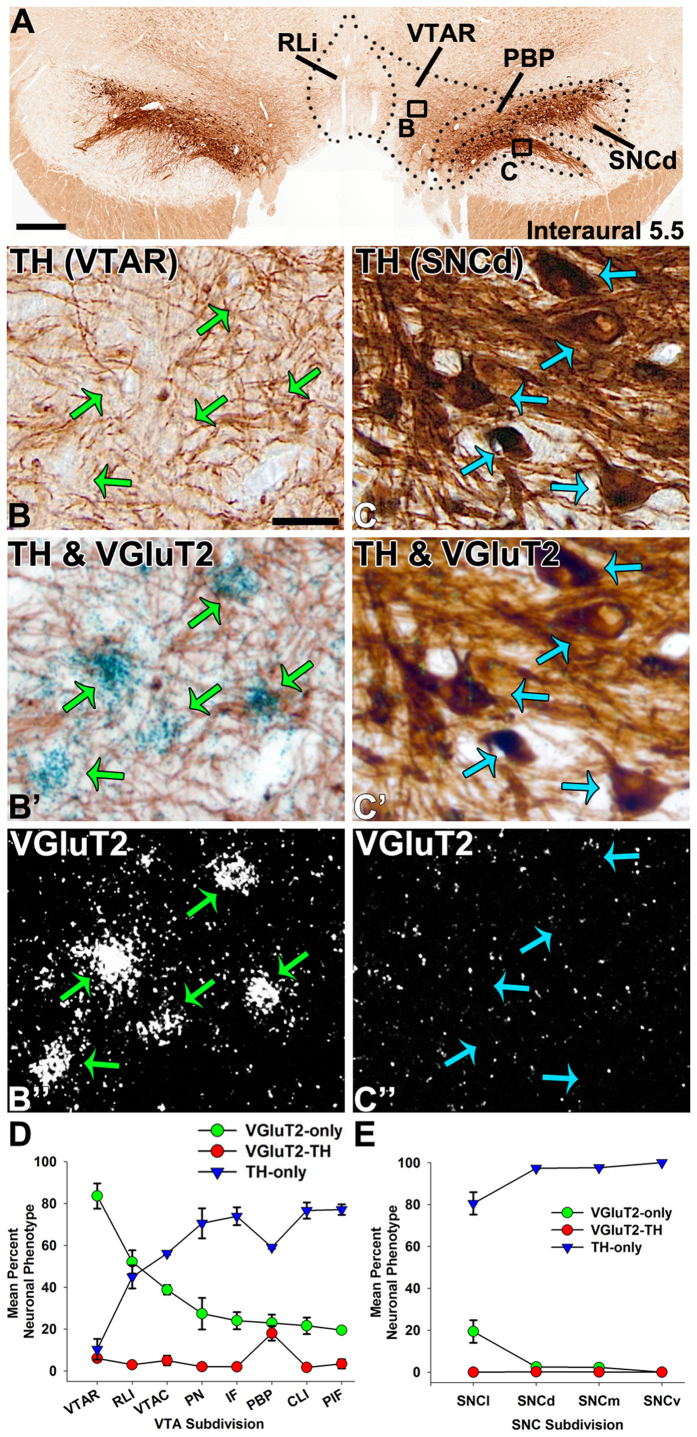
Presence of VGluT2 neurons intermingled with TH neurons in the marmoset VTA (anterior aspect) and SNC. (**A**) Coronal section showing TH immunolabeling (brown) at the level of the VTAR (rostral ventral tegmental area subdivision) (interaural 5.5). (**B-C”)**. Boxed areas in A at higher magnification showing TH immunolabeled neurons (brown cells) or cells expressing VGluT2 mRNA (clusters of grains seen as blue in brightfield, (**B’)** or white in darkfield, (**B”**). (**B-B”)**. VTAR cells expressing VGluT2 mRNA and lacking TH (VGluT2-only neurons; green arrows). (**C-C”)**. SNCd (dorsal division of the substantia nigra pars compacta) cells lacking VGluT2 mRNA, but containing TH (blue arrows; TH-only neurons). Scale bar in A = 250 μm, scale bar in B = 25 μm and applies to B-B” and C-C”. (**D,E)**. Distribution of VGluT2-only neurons (green circles), TH-only neurons (blue triangles), or VGluT2-TH neurons (red circles) within different aspects of the VTA (**D**) or the SNC (**E**). (**D**) Within the VTA, the VTAR contains mainly VGluT2-only neurons whereas the RLi (rostral linear nucleus) and VTAC (caudal ventral tegmental area subdivision) contain almost equivalent proportions of VGluT2-only and TH-only neurons. The IF (interfascicular nucleus), PN (paranigral nucleus), PIF (parainterfascicular nucleus), and CLi (caudal linear nucleus) contain mostly TH-only neurons and fewer VGluT2-only neurons. The PBP (parabrachial pigmented nucleus) contains mostly TH-only neurons and uniquely exhibits dual VGluT2-TH neurons. (**E**) Within the SNC the VGluT2-only neurons are confined to the SNCl (substantia nigra pars lateralis). Data in (**D,E**) are mean ± SEM percent of the neuronal phenotype within each subdivision per analyzed section.

**Figure 2:**
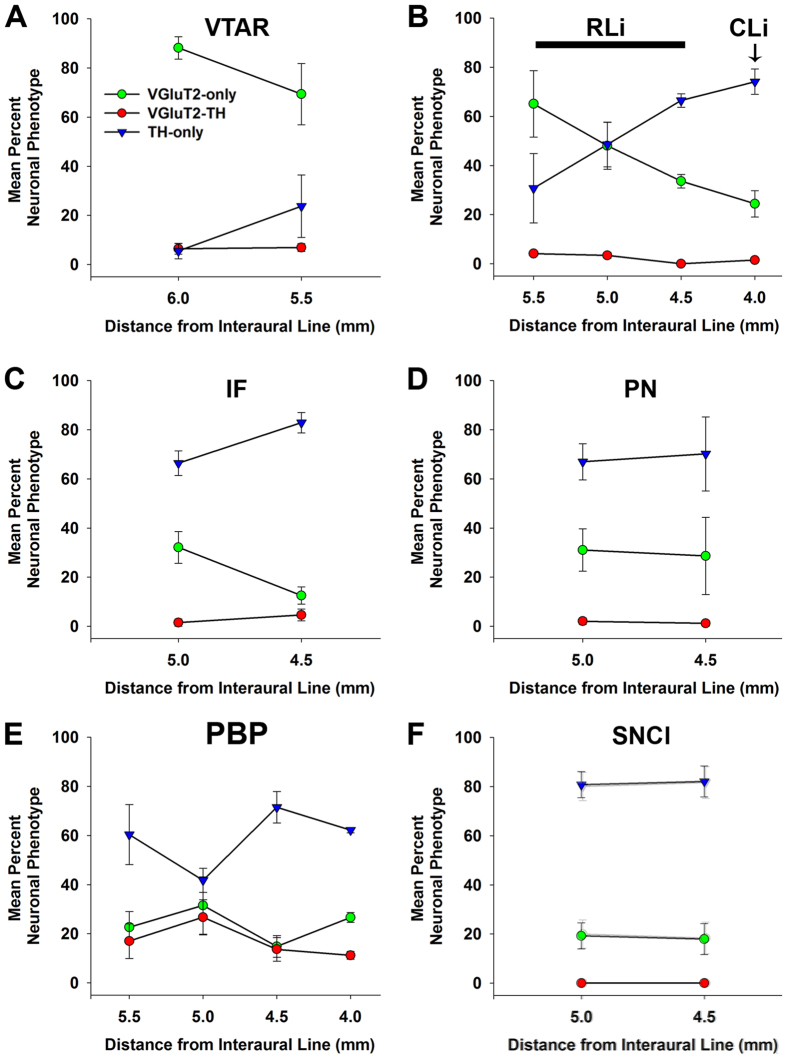
Changes in prevalence of neuronal phenotypes within marmoset VTA and SNC subdivisions across the anteroposterior plane. (**A–F)** Distribution of VGluT2-only neurons, (green circles), TH-only neurons (blue triangles), and VGluT2-TH neurons (red circles) within specific midbrain subdivisions, (**A**) Decreasing trend of VGluT2-only neurons and increasing trend of TH-only neurons in the VTAR along the anteroposterior axis. (**B**) Decreasing trend of VGluT2-only neurons and increasing trend of TH-only neurons in the RLi along the anteroposterior axis that continues into the CLi. (**C**) Decreasing trend of VGluT2-only neurons and increasing trend of TH-only neurons in the IF along the anteroposterior axis. (**D**) Neuronal phenotypes do not differ along the anteroposterior axis of the PN. (**E**) VGluT2-TH neurons are a major subpopulation in the PBP, especially at interaural 5.0 where the prevalence of all neuronal phenotypes is similar. (**F**) No change in prevalence of VGluT2-only neurons in the SNCl across the anteroposterior plane. Data are mean ± SEM percent of the neuronal phenotype within each subdivision per analyzed section.

Within the rostral VTA subdivision of the VTA (VTAR), we found mostly VGluT2-neurons that did not co-express TH (VGluT2-only neurons) (Fig. 1A,B,D). These VGluT2-only neurons constituted over 80% of all labeled VTAR neurons (Table 1). In contrast to the prevalence of VGluT2-only neurons, approximately 10% of the labeled VTAR neurons expressed TH but lacked VGluT2 (TH-only neurons). The prevalence of TH-only neurons slightly increased along the anteroposterior axis (Fig. 2A). The abundance of VGluT2-neurons within the VTAR differed from the neighboring SNC, which was dominated by TH-only neurons (Fig. 1C,E).

**Figure 3:**
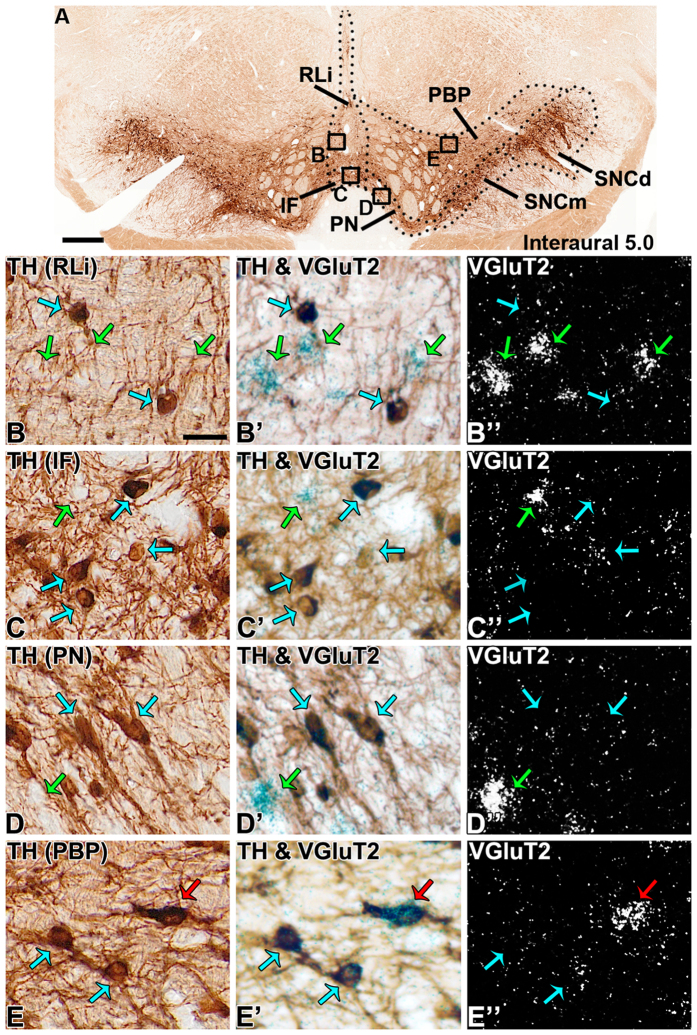
VGluT2 neurons in the central portions of the marmoset VTA and SNC. (**A**) VTA coronal section showing TH-expression at a central anteroposterior portion of the VTA and SNC (interaural 5.0). (**B-E”)** Higher magnification of boxed areas in A. The RLi (**B-B”**) exhibits a mixture of VGluT2-only neurons (green arrows) and TH-only neurons (blue arrows) while the IF (**C-C”**) and PN (**D-D”**) exhibit mostly TH-only neurons with fewer VGluT2-only neurons. The central PBP (**E-E”**; at interaural 5.0) contains VGluT2-only, TH-only neurons and VGluT2-TH neurons (red arrow). Scale bar in A = 250 μm, scale bar in B = 25 μm and applies to B-B”, C-C”, D-D”, and E-E”.

**Figure 4:**
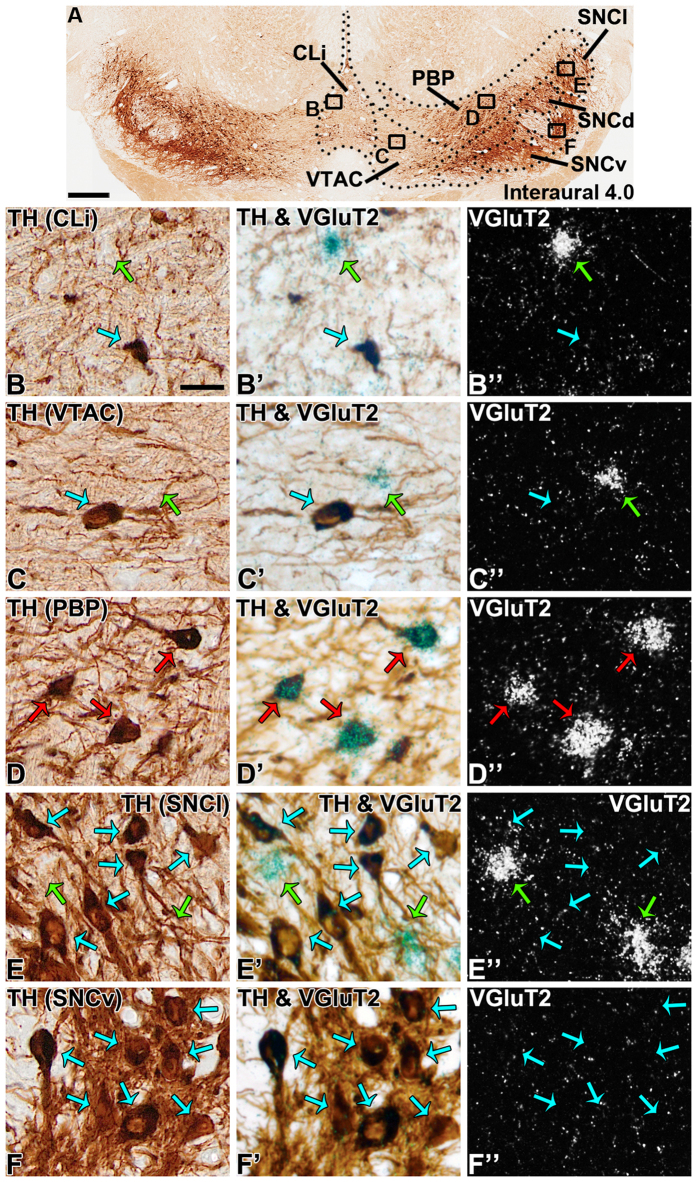
VGluT2 neurons in the caudal portions of the marmoset VTA and SNC. (**A**) Coronal section showing TH-expression at the level of the CLi (interaural 4.0). (**B-F”)** Higher magnification of boxed areas in A. (**B-C”)** Detection of VGluT2-only and TH-only neurons in the CLi (**B**) and VTAC (**C**). (**D)** Frequent detection of dual VGluT2-TH neurons (red arrows) in the caudal PBP. (**E**) Detection of VGluT2-only neurons and TH-only neurons in the SNCl. (**F**) Lack of detection of VGluT2 neurons within the SNCv. Scale bar in A = 250 μm, scale bar in B = 25 μm and applies to B-B”, C-C”, D-D”, E-E”, and F-F”.

Within the rostral linear nucleus (RLi; most medial aspect of the VTA), approximately half of the neurons were VGluT2-only neurons or TH-only neurons (Table 1) (Figs 1D and 3A,B). The prevalence of VGluT2-only neurons within the RLi decreased as the prevalence of TH-only neurons increased along the anteroposterior axis (interaural 5.5 to 4.5) (Fig. 2B), such that in the most anterior aspect of the RLi (interaural 5.5), 65.10% ± 13.50% of the counted RLi neurons were VGluT2-only neurons and 30.72% ± 14.09% were TH-only neurons. In contrast to the anterior RLi, approximately one-third of the neurons in the posterior aspect of the RLi (interaural 4.5) were VGluT2-only neurons (33.59% ± 2.76%) and two-thirds were TH-only neurons (66.41% ± 2.76%). The decreasing prevalence of RLi VGluT2-only neurons and increasing prevalence of TH-only neurons continued into the contiguous caudal linear nucleus (CLi; interaural 4.0), which contained near to one-quarter of VGluT2-only neurons and three-quarters of TH-only neurons (Table 1) (Figs 2B and 4A,B). Despite the decreased prevalence of VGluT2-only neurons from the anterior to the posterior aspects of the VTA, a small population of VGluT2-only neurons was observed in the caudal VTA subdivision (VTAC; Table 1) and the rest were TH-only neurons (Figs 1D and 4C). Dual VGluT2-TH neurons were rarely observed in the VTAC.

Within the interfascicular nucleus (IF), a medioventral subdivision of the VTA, approximately one-quarter of the labeled neurons were VGluT2-only neurons and about three-quarters were TH-only neurons (Table 1) (Figs 1D and 3C). Similar to the RLi, the frequency of VGluT2-only neurons decreased along the anteroposterior axis of the IF, while the frequency of TH-only neurons increased (Fig. 2C). We found that within the paranigral nucleus (PN) of the VTA, the VGluT2-only neurons constituted near to one-third of the total labeled neurons and the rest were TH-only neurons (Table 1), and this prevalence was maintained along the anteroposterior axis (Figs 1D,2D and 3D). Within the parainterfascicular nucleus (PIF) of the VTA, about one-fifth of neurons were VGluT2-only neurons and near to three-quarters were TH-only neurons (Table 1) (Fig. 1D).

**Figure 5:**
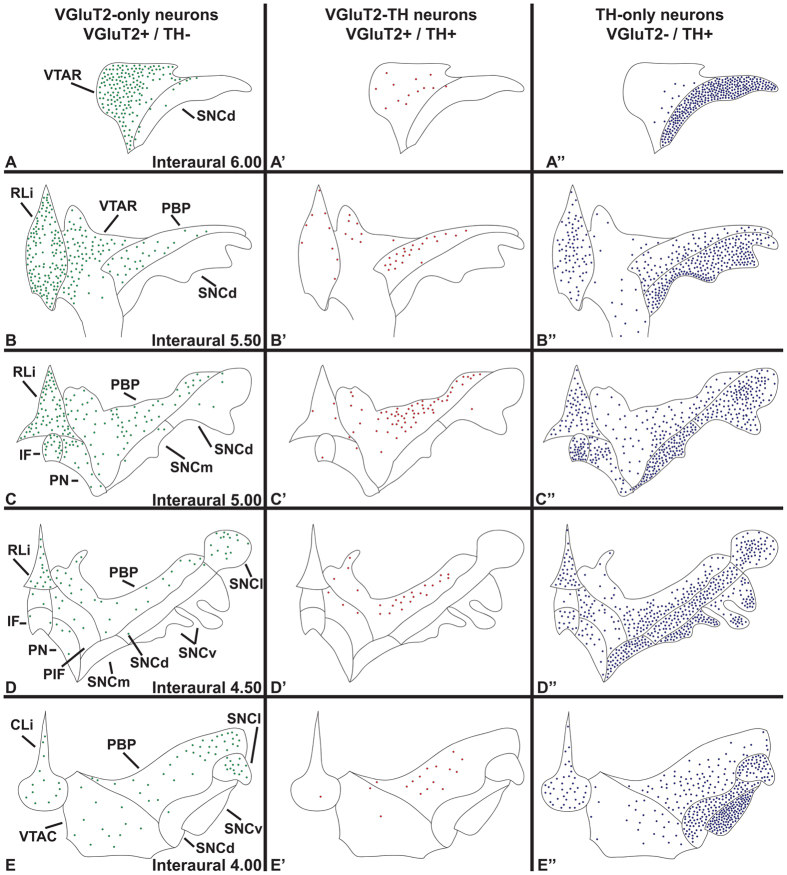
Summary diagram of the cellular heterogeneity within the marmoset VTA and SNC. Distribution of VGluT2-only neurons, VGluT2-TH neurons, and TH-only neurons within the subdivisions of the marmoset VTA and SNC. (**A–E**) The VGluT2-only neurons are present throughout the rostrocaudal levels of each subdivision of the VTA with a decreasing gradient of concentration along the anteroposterior axis. In the VTA, VGluT2-only neurons are most frequent in the VTAR and RLi, and in the SNC are exclusive to the SNCl. (**A’-E’**) VGluT2-TH neurons are largely found in the PBP, however, they are present in all VTA subdivisions. (**A”-E”**) The TH-only neurons are present throughout the rostrocaudal levels of each VTA and SNC subdivision. Each panel represents the average number of labeled neurons found in each subdivision.

We determined that within the PBP of the VTA about one-quarter of the labeled neurons were VGluT2-only neurons, and more than half were TH-only neurons (Table 1) (Fig. 1D). Among all marmoset VTA subdivisions, the PBP had the highest frequency of dual VGluT2-TH neurons, representing 18.03 ± 3.47% of all counted PBP neurons (Figs 3E,4D and 5). In contrast, the prevalence of VGluT2-TH neurons was less than 7% in each of the other subdivisions of the VTA (Fig. 1D). Within the total population of VGluT2 expressing neurons 41.99% were dual VGluT2-TH neurons, and within the total population of TH-neurons 23.45% were dual VGluT2-TH neurons (Table 1). We detected the greatest numbers of dual VGluT2-TH neurons in the central portion of the PBP (at interaural level 5.0) where the proportions of VGluT2-only, TH-only and dual VGluT2-TH neurons were similar (Fig. 2E).

The marmoset SNC was dominated by TH-only neurons (Figs 1E and 5), which were prominent within the dorsal subdivision (SNCd), ventral division (SNCv), and pars medialis subdivision of the SNC (SNCm) (Table 2). We detected VGluT2-only neurons intermingled with TH-only neurons in the pars lateralis subdivision of the SNC (SNCl) (Table 2). Within the SNCl, the VGluT2 neurons represented approximately one-fifth of the labeled neurons (Figs 1E and 4E). The proportions of both VGluT2-only and TH-only neurons within the SNCl were maintained over the anteroposterior axis (Fig. 2F). We rarely found dual VGluT2-TH neurons within the SNC (Fig. 1E), and did not detect VGluT2-expressing neurons within the SNCv (Fig. 4F).

### Detection and distribution of VGluT2-neurons, TH-neurons and dual VGluT2-TH neurons within the subdivisions of the human VTA and SNC

Detection of neurons in the human VTA has remained challenging due to limited access to brain tissue and in most instances, receipt of brain tissue that has endured substantial loss of mRNA, the integrity of which is necessary for the cellular identification of neurons expressing VGluT2 mRNA. To optimize the cellular detection of both TH-protein and VGluT2-mRNA, human brains were maintained in ice and were immediately fixed by perfusion instead of by fixed immersion into a fixative solution. The optimal molecular and cellular preservation of the human brain tissue allowed us to combine immunodetection of TH (to establish the boundaries and subdivisions of the VTA and SNC) and radioactive *in situ* hybridization to identify neuronal expression of transcripts encoding VGluT2 mRNA.

**Figure 6:**
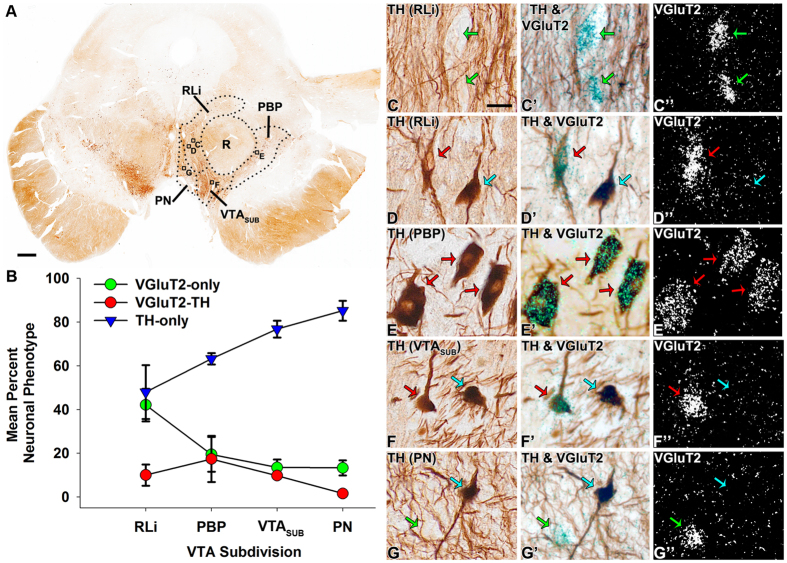
Prevalence of neuronal phenotypes within human VTA subdivisions. (**A**) Coronal section showing TH-expression in the human VTA. (**B**) Distribution of VGluT2-only neurons (green circles), TH-only neurons (blue triangles) and dual VGluT2-TH neurons (red circles) within the different subdivisions of the human VTA. A mixture of both VGluT2-only and TH-only neurons is present in the RLi. The highest prevalence of dual VGluT2-TH neurons is present in the PBP. The PN and the VTA_SUB_ are mostly populated by TH-only neurons. Data are mean ± SEM percent of the neuronal phenotype within each subdivision per analyzed section. (**C-G”)** Neuronal phenotypes detected within the human VTA subdivisions. C-D”. RLi detection of both VGluT2-only neurons (green arrows in C-C”) and dual VGluT2-TH neurons (red arrows in D-D”). E-E’. PBP detection of dual VGluT2-TH neurons (red arrows). (**F-F”**) VTA_SUB_ detection of TH-only neurons (blue arrows) and dual VGluT2-TH neurons (red arrows). (**G-G”**) PN detection of TH-only neurons (blue arrow) and VGluT2-only neurons (green arrows). Scale bar in A = 250 μm, scale bar in C = 25 μm and applies to C-C”, D-D”, E-E”, F-F”, and G-G”.

As in rodents and in nonhuman primates, we detected neurons expressing VGluT2 mRNA intermingled with TH-neurons in the human VTA and SNC. We found that the human VTA and SNC shared a distribution of VGluT2 neurons similar to that observed in the marmoset. Within the human VTA, we detected the highest prevalence of VGluT2-only neurons in the RLi (42.04% ± 7.44% of RLi neurons; Table 3). In contrast to the infrequency of dual VGluT2-TH neurons in the marmoset RLi (less than 3% of neurons), we found that dual VGluT2-TH neurons in the human RLi represented one-tenth of the labeled RLi neurons. As in the marmoset, the TH-only neurons in the human RLi constituted approximately half of the RLi neurons (Fig. 6A–D).

In common with the marmoset PBP, the human PBP contained VGluT2-only, TH-only and dual VGluT2-TH neurons. We found that in the human PBP about 60% of the labeled neurons were TH-only neurons and 20% were VGluT2-only neurons (Table 3). In common with the marmoset findings, we detected within the human PBP the highest prevalence of dual VGluT2-TH neurons (Fig. 6B,E). We further estimated that within the human PBP the dual VGluT2-TH neurons represented 39.45% of the total population of VGluT2-expressing neurons and 18.19% of the total population of TH-expressing neurons (Table 3).

The VGluT2-only, TH-only and dual VGluT2-TH neurons were also detected in the VTA region located between the PBP and RLi, known as the ‘VTA subdivision’ of the VTA (VTA_SUB_)^28^ (Fig. 6F). We found that within the total population of VTA_SUB_ neurons over three-quarters of these neurons were TH-only neurons, 10% were VGluT2-only neurons and 10% were VGluT2-TH neurons (Table 3). However, within the total population of VGluT2-expressing neurons, 41.77% were dual VGluT2-TH neurons and within the total population of TH-neurons 11.32% were dual VGluT2-TH neurons. In contrast to the high frequency of dual VGluT2-TH neurons detected in the PBP and the VTA_SUB_, the PN neurons were largely segregated into VGluT2-only neurons and TH-only neurons, and dual VGluT2-TH neurons were rarely observed (Table 3) (Fig. 6G).

**Figure 7:**
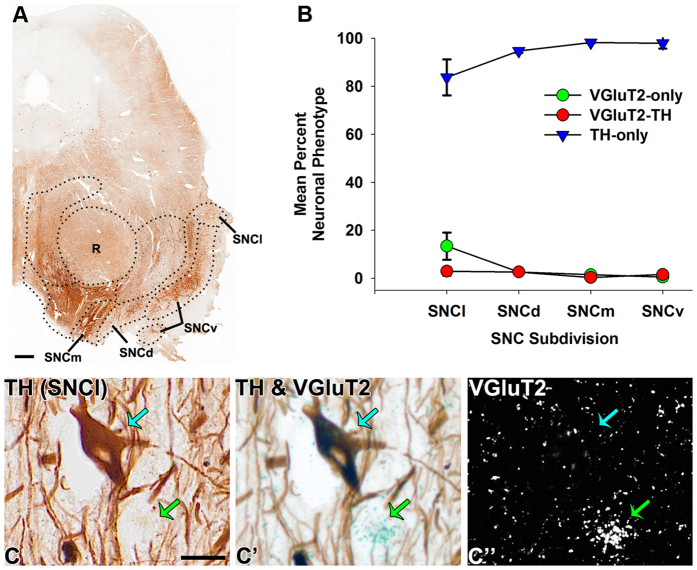
Prevalence of neuronal phenotypes within human SNC subdivisions. (**A**) Coronal section showing TH-expression in the human SNC. (**B**) Distribution of VGluT2-only neurons (green circles), TH-only neurons (blue triangles) and dual VGluT2-TH neurons (red circles) within the different subdivisions of the human SNC. The SNC has mostly TH-only neurons within all subdivisions and infrequent VGluT2 neurons that are confined to the SNCl. Data are mean ± SEM percent neuronal phenotype within each subdivision per analyzed section. (**C**) Detection of a TH-only neuron (blue arrow) and a VGluT2-only neuron (green) within the SNCl. Scale bar in A = 250 μm, scale bar in C = 250 μm and applies to C-C”.

**Figure 8:**
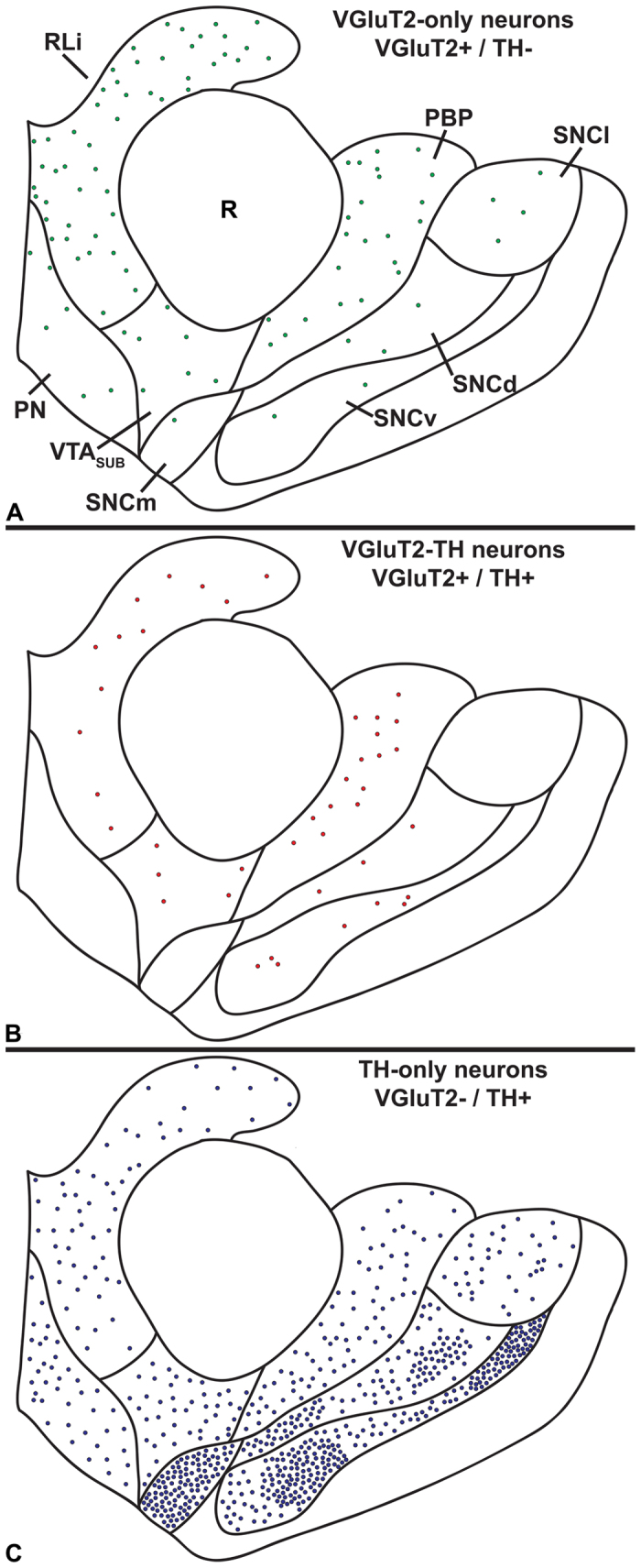
Summary diagram of the cellular heterogeneity within the human VTA and SNC subdivisions. Distribution of TH-only neurons, dual VGluT2-TH neurons, and VGluT2-only neurons within each subdivision of the human VTA and SNC. (**A**) VGluT2-only neurons are present throughout the VTA subdivisions but they are most frequent in the RLi. Within the SNC, pars lateralis (SNCl) contains the most VGluT2-only neurons. (**B**) Dual VGluT2-TH neurons are most prevalent in the PBP and RLi, they are also found within the VTA_SUB_, but they are rarely found in the PN.(**C**) TH-only neurons are abundant within all subdivisions of the SNC. Each panel represents the average number of labeled neurons found in seven to twelve sections per midbrain subdivision.

As in the marmoset SNC, the human SNCd, SNCv, and SNCm subdivisions of the SNC were dominated by TH-only neurons (Table 4). In addition, we detected VGluT2 neurons in the human SNCl that in their vast majority did not co-express TH (Table 4) (Figs 7 and 8). While we did not detect VGluT2-neurons in the marmoset SNCv, a small population of VGluT2-neurons was identified in the human SNCv.

## Discussion

The conservation of VTA and SNC dopamine neurons from mouse to human has long been established^29^,^30^,^31^. However, recent findings from mouse and rat studies have shown that in addition to dopamine neurons, the rodent VTA and SNC have glutamate neurons^13^,^14^,^32^,^33^. These rodent glutamate neurons are diverse in their biochemical composition, neuronal signaling, and target areas^15^,^34^. Furthermore, rodent VTA glutamate neurons play roles in aversion^24^,^35^,^36^ or reward^25^. Here, we provide evidence for the presence of glutamate neurons within the VTA and SNC in nonhuman primates and humans. These neurons may play similar roles as those ascribed to their rodent counterpart. As such, the human VTA glutamate neurons may contribute to reward and aversion, and VTA-related illnesses, such as depression or addiction. In this regard, a human VTA microarray gene expression screening has shown that expression of VGluT2 mRNA is higher in VTA samples from individuals with a history of nicotine abuse, compared with control subjects^37^.

Studies in nonhuman primates have shown that putative dopamine neurons in the VTA and SNC, identified by their electrophysiological properties, are activated in response to unpredicted rewards, shift their activation to predictors of reward following cue-reward learning, and omission of an expected reward depresses putative dopamine neuron activity^38^,^39^,^40^,^41^,^42^,^43^,^44^,^45^,^46^,^47^,^48^,^49^. These different firing patterns reflective of reward prediction have been a cornerstone of theories regarding how these dopamine neurons participate in everyday decision making, as well as a variety of human disorders involving dopamine signaling, such as addiction, depression, movement disorders, impulse control disorders, and schizophrenia^4^,^5^,^7^,^8^,^50^,^51^,^52^,^53^,^54^,^55^,^56^,^57^,^58^,^59^. Importantly, studies in mice and rats have confirmed the dopamine nature of reward prediction signaling by midbrain dopamine neurons^26^,^60^,^61^,^62^,^63^. Additional studies from electrophysiological recordings in nonhuman primates have shown that subsets of midbrain putative dopamine neurons are responsive to punishers and their predictors^64^. Taken together, the nonhuman primate electrophysiological findings have provided evidence for functional heterogeneity among putative dopamine neurons, which may reflect diversity in their biochemical composition or their neuronal connectivity. However, information on nonhuman primate midbrain neuronal phenotypes and their neuronal connectivity are very limited. In contrast, substantial progress has been made recently in elucidating a great diversity among the midbrain dopamine neurons and among their intermixed nondopamine neurons, and the involvement of these different types of neurons in a variety of motivated behaviors^15^,^19^,^22^,^24^,^27^,^35^,^65^,^66^,^67^,^68^.

Recent rat and mouse studies have shown that VTA glutamate neurons establish local and long-range connections that participate in different types of synapses and behaviors. At the local and synaptic levels, the VTA glutamate neurons establish multiple asymmetric synapses on single neighboring dopamine neurons; these synapses mediate glutamate-receptor-dependent firing of some of the dopamine neurons that innervate the nucleus accumbens^25^,^69^. At the VTA local level, activation of glutamate neurons mediates reward that depends on activation of local glutamatergic receptors^25^. While most of the mouse and rat VTA-glutamate neurons lack both dopaminergic and GABAergic markers, within the midline aspects of the VTA there are subpopulations of glutamate neurons that co-express molecules for the synthesis and vesicular accumulation of either dopamine^14^,^32^,^70^ or GABA^15^. Emerging evidence suggests that each subpopulation of glutamate-dopamine neurons, glutamate-GABA neurons, and glutamate-only neurons target selective neuronal populations within different brain structures. For instance, the fibers from VTA glutamate-dopamine neurons establish asymmetric excitatory synapses on dendritic spines of medium spiny neurons (MSNs) within the nucleus accumbens^34^, and the optical activation of these glutamate-dopamine fibers excites both MSNs^12^,^71^ and cholinergic interneurons^72^. A recent study has demonstrated that in the nucleus accumbens individual axons from glutamate-dopamine neurons has the capability to release dopamine and glutamate from mutually exclusive microdomains^34^. As such, a single glutamate-dopamine axon may provide to accumbal MSNs or cholinergic interneurons both fast excitatory signaling (via glutamate) and prolonged modulatory signaling (via dopamine). In addition to the nucleus accumbens, the dual VTA glutamate-dopamine neurons innervate the medial prefrontal cortex^14^,^73^, where they target parvalbumin-expressing interneurons, and contribute to perseverative behaviors^73^. In contrast to the participation of dual glutamate-dopamine neurons in a mesocortical pathway, the glutamate-GABA neurons provide the major input from the VTA to the lateral habenula^15^. Individual axon terminals from these mesohabenular glutamate-GABA neurons establish a balance of excitatory and inhibitory control on glutamate neurons within the lateral habenula^15^. Lateral habenula activation of fibers from VTA glutamate-GABA neurons induces aversion, which is mediated by activation of local glutamatergic receptors^15^. Regarding VTA glutamate-only neurons, a population of nucleus accumbens parvalbumin-interneurons constitutes a major target of fibers from the VTA glutamate-only neurons. These fibers establish multiple asymmetric synapses on single parvalbumin interneurons at the level of both dendrites and cell bodies^36^. The activation of these fibers synapsing on parvalbumin-interneurons results in the release of GABA onto accumbal MSNs, and the promotion of aversion^36^.

The recent findings detailed above indicate that the rodent midbrain glutamate neurons participate in different aspects of reward or aversion. In addition, some of these glutamate neurons have the capability to use both glutamate and dopamine, or glutamate and GABA as signaling molecules, and they target brain structures implicated in several brain disorders. Here, we report that as in mice and rats, the midbrain dopamine neurons of both nonhuman primates and humans are intermixed with glutamate-only and dual glutamate-dopamine neurons. However, whereas the mouse RLi is dominated by glutamate-only neurons (>80% of neurons) and the rat RLi contains approximately 60% glutamate-only neurons^14^,^33^, a lower prevalence of glutamate-only neurons is present in the marmoset RLi (~50% of RLi neurons) and human RLi (40% of RLi neurons). However, it is possible that the prevalence of human RLi glutamate neurons is increased in more anterior sections than those analyzed in the present study, as glutamate neurons dominate the most anterior portions of the mouse, rat, and marmoset RLi^14^,^33^. The decreased prevalence of glutamate-only neurons within the RLi from mouse to human is concomitant with an increase of the prevalence of dopamine-only neurons within the RLi from the mouse to human. We had previously shown that a large proportion of the rat RLi TH-expressing neurons lack the dopamine transporter (for reuptake of dopamine) and the vesicular monoamine transporter 2 (VMAT2; for accumulation of dopamine into synaptic vesicles)^70^,^74^. In contrast, nearly all rat dopamine neurons residing in the lateral VTA neighboring to the SNC (lateral PBP) co-express both the dopamine transporter and VMAT2^70^. A similar lateral-to-medial decreasing gradient of dopamine transporter and VMAT2 expression has been observed from the PBP to the RLi in the human VTA^75^, suggesting that several subtypes of dopamine neurons are also present across species.

Regarding the detection and distribution of glutamate-only and dopamine-only neurons, we found that within the most anterior aspects of the marmoset VTA, where VTAR is present, the glutamate-only neurons are the major neuronal population of neurons in both the VTAR and RLi. As in mice and rats, the VTA of nonhuman primates have a decreasing gradient of glutamate-only neurons and an increasing gradient of dopamine-only neurons across the anteroposterior axis within the VTAR, RLi, and IF. However, the marmoset VTAC is an exception to these cross-species gradients, as it contains a large proportion of glutamate-only neurons (~40%), though the total number of neurons is comparatively low. Compared to mice and rats, several nuclei of the marmoset VTA (IF, PN, and PIF, CLi) show a lower proportion of glutamate-only and glutamate-dopamine neurons, and a higher prevalence of dopamine-only neurons. There are also some anatomical differences between species that are likely to contribute to differences in the prevalence of midbrain neuronal phenotypes. For instance, both VTAC of marmosets and VTA_SUB_ of humans are not present in rodents.

Regarding the detection and distribution of dual glutamate-dopamine neurons, we found that in the marmoset and human VTA these neurons are most prevalent within the lateral parts of the VTA, specifically the PBP. This prevalence and distribution are in contrast to that present in the mouse and rat VTA, in which the dual glutamate-dopamine neurons have their highest prevalence in the medial aspects of the VTA^33^. While we also detected dual glutamate-dopamine neurons in the human RLi and VTA_SUB_, these neurons are infrequent in the marmoset RLi, and undetectable in the human or marmoset PN. Thus, the presence of dual glutamate-dopamine neurons is a characteristic shared by the rat RLi and human RLi, but not by the marmoset RLi. The glutamate neurons of the rat PBP and PN are exclusively located within their most medial portions, which contain a high prevalence of dual glutamate-dopamine neurons^13^,^14^. In contrast to the prevalence of dual glutamate-dopamine neurons in the medial aspects of the PBP and PN in the rat^13^,^14^, the marmoset and human PBP contain glutamate-dopamine neurons in both of their medial and lateral aspects. In this context, in primates the firing patterns of putative midbrain dopamine neurons related to reward prediction, reward, aversion, or value have largely been identified in the lateral parts of the midbrain, which include the lateral PBP^43^,^44^,^47^,^48^,^76^,^77^,^78^. Thus some of these recorded neurons within the lateral PBP may correspond to dual glutamate-dopamine neurons, as the primate lateral PBP contains the largest population of dual glutamate-dopamine neurons, suggesting glutamate-dopamine neurons might participate in reward-prediction signaling.

From our findings, we suggest that the observed activation of medial and lateral portions of the human dopaminergic midbrain measured by imaging studies is likely to include the participation of glutamate-only and glutamate-dopamine neurons intermingled with the dopamine-only neurons. Imaging studies have shown functional MRI signal changes in the medial, lateral, or both parts of the human VTA in response to a variety of motivation-related stimuli, including the consumption of drugs of abuse^79^,^80^,^81^,^82^,^83^,^84^,^85^,^86^,^87^. Portions of the human medial VTA, which appear to include the RLi and PN, exhibit increased reactivity during threat processing^88^,^89^, and pain anticipation^90^. Although the cellular phenotypes of the medial VTA that mediate the observed increased reactivity cannot be determined by imaging, based on our findings of human cellular characterization within the RLi and PN (Fig. 8), it is possible that, in addition to human VTA dopamine neurons, the VTA glutamate neurons located in the medial aspects of the human VTA participate in the documented increased reactivity within the VTA during threat processing or pain anticipation. As in the human medial VTA, glutamate neurons are also present in the mouse and rat medial VTA. Regarding medial VTA glutamate neurons, we had previously identified within the mouse RLi and PN a subset of glutamate neurons that innervate the lateral habenula, and are involved in aversive conditioning^24^. Regarding medial VTA dopamine neurons, *in vivo* recordings in the rat have shown that dopamine neurons of the rat PN exhibit elevated firing rates in response to noxious stimuli^91^. Thus, we suggest that both glutamate and dopamine neurons of the human medial VTA are likely to play roles in aversive processing.

Human imaging studies have shown that the ventromedial SNC exhibits activity reflective of reward prediction while the dorsolateral SNC exhibits activity reflective of aversion prediction^92^. The neuronal activity of the dorsolateral SNC has also been shown to exhibit increased firing rates in response to both rewarding and aversive stimuli while ventromedial SNC neurons exhibit increased firing rates only to rewarding stimuli^64^. These human and nonhuman primate functional findings suggest functional heterogeneity within the SNC that may reflect, in part, neuronal heterogeneity within the SNC. In this regard, we present evidence that glutamate neurons are confined to the SNCl, which is the most dorsolateral SNC subdivision in humans and marmosets, and a similar distribution is observed in the rat^32^. Given that the VTA glutamate neurons are involved in aversive conditioning^24^, it is conceivable that the SNCl glutamate neurons have a similar behavioral function. However, further research will be necessary to determine the distinct influences of each SNCl neuronal phenotype in motivated behavior.

In summary, we provide evidence that 1) glutamate-only, dopamine-only, and dual glutamate-dopamine neurons are present within the marmoset and human VTA, the prevalence of which depends upon the VTA subdivision; and 2) a population of glutamate-only neurons is present within the SNCl subdivision, while the SNC is otherwise dominated by dopamine-only neurons. The discovery that glutamate-only, dopamine-only, and dual glutamate-dopamine neurons are differentially distributed within the midbrain dopamine systems from rodents to humans will allow further exploration of the specific roles that these different neurons play in behavior. Specifically, the advances made in identifying distinct neuronal phenotypes, together with the analysis of their functions by application of optogenetic methodologies in transgenic rodents, where cell-specific manipulations can be made, provides a foundation for investigating human-related midbrain-associated illnesses. Findings from the present studies may provide useful information on determining the unique contributions of individual VTA neuronal phenotypes in disorders such as addiction. Within this context, recent advancements in magnetic strength used in imaging studies may allow the evaluation of the functions of discrete VTA subdivisions in humans^93^.

## Methods

### Nonhuman primate tissue collection

All animal procedures were performed in accordance with National Institutes of Health Guidelines, and approved by the National Institute of Neurological Disorders and Stroke Animal Care and Use Committee. Three male marmosets were deeply anesthetized with ketamine hydrochloride (10 mg/kg, iv) followed by sodium pentobarbital (15 mg/kg, iv). Marmosets were perfused transcardially with 0.9% NaCl containing 2% sodium nitrite (800 ml) to flush blood from the vascular system, followed by 4% paraformaldehyde in 0.1 M phosphate buffer (PB, pH 7.0, 1 L). Brains were extracted, stored in 4% paraformaldehyde overnight at room temperature, and on the next day changed to a 16% sucrose/1% paraformaldehyde solution in PB at 4 °C. Brains were frozen in isopentane and stored at −80 °C until sectioning. The VTA was coronally sectioned (12 μm) in a cryostat and stored in 24 tubes containing 30% sucrose/30% polyethylene glycol in RNAse-free PB.

### Human tissue collection

Two subjects were processed for autopsy in Saint Borbála Hospital, Tatabánya, Department of Pathology. Informed consent was obtained for the use of brain tissue and for access to medical records for research purposes. Tissue was obtained and used in a manner compliant with the Declaration of Helsinki. All procedures were approved by the Regional and Institutional Committee of Science and Research Ethics of Scientific Council of Health (ETT TUKEB 31443/2011/EKU (518/PI/11)). Brains were extracted between 2.5 to 3.5 hours post mortem. Both internal carotid and vertebral arteries were cannulated, and the brains were perfused first with physiological saline (1.5 liters for 30 minutes) containing 5 ml of heparin, followed by a fixative solution containing 4% paraformaldehyde, 0.05% glutaraldehyde and 0.2% picric acid in 0.1 M PB (pH 7.4, 4–5 liters for 1.5–2 hours). After perfusion the brains were postfixed in the same fixative solution overnight, but without glutaraldehyde^94^. Brains were then stored in 0.1 M PB with sodium azide while shipping to NIDA facilities. Next, brains were stored in 18% sucrose at 4 °C, frozen in isopentane, and stored at −80 °C until sectioning. The VTA was coronally sectioned (12 μm) in a cryostat and stored in 180 tubes containing 30% sucrose/30% polyethylene glycol in water. Subject 1 was a 55 year old male and cause of death was pulmonary embolism with acute cor pulmonale. Subject 2 was a 70 year old female and cause of death was subsequent ST segment elevation myocardial infarction of the inferior wall. Subjects had no history of neurological disease.

### *In situ* hybridization and immunohistochemistry

Sections were incubated for 10 min in PB containing 0.5% Triton X-100, rinsed two times for 5 min each with PB, treated with 0.2 N HCl for 10 min, rinsed two times for 5 min each with PB, and then acetylated in 0.25% acetic anhydride in 0.1 M triethanolamine, pH 8.0, for 10 min. Sections were rinsed two times for 5 min each with PB and postfixed with 4% PFA for 10 min. Before hybridization and after a final rinse with PB, the free-floating sections were incubated in hybridization buffer (50% formamide, 10% dextran sulfate, 5X Denhardt’s solution, 0.62 M NaCl, 50 mM DTT, 10 mM EDTA, 20 mM PIPES, pH 6.8, 0.2% SDS, 250 μg/ml salmon sperm DNA, 250 μg/ml tRNA) for 2 h at 55 °C. Sections were hybridized for 16 h at 55 °C in hybridization buffer containing [35^S^]- and [33^P^]-labeled single-stranded antisense of marmoset VGluT2 (nucleotides 674–2422; GenBank accession number XM_002755104.3) or human VGluT2 (nucleotides 707–4394; GenBank accession number NM_020346.1), probes at 107 cpm/ml. Sections were treated with 4 μg/ml RNase A at 37 °C for 1 h, washed with 1X SSC, 50% formamide at 55 °C for 1 h, and with 0.1X SSC at 68 °C for 1 h. After the last SSC wash, sections were rinsed with PB and incubated for 1 h in PB supplemented with 4% bovine serum albumin and 0.3% Triton X-100. This was followed by the overnight incubation at 4 °C with an anti-TH mouse monoclonal antibody (1:500; MAB 318; Millipore) for which specificity has been documented^95^. After being rinsed three times for 10 min each in PB, sections were processed with an ABC kit (Vector Laboratories). The material was incubated for 1 h at room temperature in a 1:200 dilution of the biotinylated secondary antibody, rinsed with PB, and incubated with avidin-biotinylated horseradish peroxidase for 1 h. Sections were rinsed and the peroxidase reaction was then developed with 0.05% 3, 3-diaminobenzidine-4 HCl and 0.03% hydrogen peroxide. Free-floating sections were mounted on coated slides. Slides were dipped in Ilford K.5 nuclear tract emulsion (Polysciences; 1:1 dilution in double distilled water) and exposed in the dark at 4 °C for 4 weeks before development.

### Data analysis of cellular subpopulations

Sections were photographed with bright-field, darkfield, and epiluminescence microscopy using an Olympus VS-100 microscope. Single- and double-labeled neurons were observed within each traced region at high power (20X objective lens) and marked electronically. Subdivisions of the marmoset VTA were traced according to a marmoset brain atlas^96^. At anteroposterior levels where the rostral VTA subdivision was present, neurons within the 3N were considered to belong to the rostral VTA. At anteroposterior levels where the VTAR subdivision was not present, neurons within the 3N were considered to belong to the PBP subdivision. The few scattered neurons identified in the ventral tegmental decussation (vtgx) were considered to belong to the RLi.

Subdivisions of the human VTA were traced based on the human brainstem atlas^97^ and descriptions of TH-expressing neuronal morphologies that define midbrain subdivisions^28^. The PBP was delineated based on the presence of TH-expressing neurons that were oriented parallel to the lateral circumference of the red nucleus. The lateral border of the PBP was the substantia nigra and dorsal border was ventral to the A8 field. We refer to the nucleus termed “VTA”^28^ as the VTA_SUB_. The VTA_SUB_ was delineated based on the presence of mediolaterally-oriented TH-expressing neurons. The PN was delineated based on small TH-expressing neurons proximal the midline. The RLi nucleus was delineated based on TH-expressing neurons that were oriented parallel to the medial circumference of the red nucleus.

TH/VGluT2 double-labeled material was analyzed using epiluminescence to increase the contrast of silver grains (neither dark-field nor bright-field optics allow clear visualization of silver grains when colocalized with high concentration of immunoproducts). A cell was considered to express VGluT2 mRNA when its soma contained concentric aggregates of silver particles above background level. A neuron was considered to express TH immunoreactivity when its soma was clearly labeled as brown. A TH-immunolabeled neuron was included in the calculation of total population of TH cells when the stained cell was at least 5 μm in diameter. The cells expressing VGluT2 mRNA, TH immunoreactivity, or both markers were counted separately. To determine cellular coexistence of VGluT2 mRNA and TH immunolabel, silver grains corresponding to VGluT2 expression were focused under epiluminescence microscopy, the path of epiluminescence light was blocked without changing the focus, and bright-field light was used to determine whether a brown neuron, expressing TH in focus, contained the aggregates of silver grains seen under epiluminescence. Labeled cells were counted three times, each time by a different observer. Pictures were adjusted to match contrast and brightness by using Adobe Photoshop (Adobe Systems).

For human data analysis, six sections from each brain were analyzed and neurons were counted only on the right hemisphere. Counters were blinded from subject-identifying information (e.g., gender, cause of death, age). Mean neuronal phenotype per subdivision was computed by averaging the percent of neurons (i.e., 10 VGluT2-only RLi neurons/20 total RLi neurons) from each section. For marmoset data analysis, twenty four sections were analyzed (11 from Dew, 8 from Turk, and 5 from Wen) and neurons were counted only on the right hemisphere, except for the midline structures (RLi and IF) which were counted in their entirety. For subdivisional analyses across the anteroposterior axis, mean neuronal phenotype per subdivision was computed by averaging the percent of neurons from one section per marmoset at each anteroposterior position (interaural 6.0 mm to 4.5 mm with a 0.5 mm interval). For subdivisional analyses without regard for anteroposterior position, mean neuronal phenotype per subdivision was computed as detailed above in the human sample analysis but for all sections in which the subdivision was identified.

## Additional Information

**Accession codes**: marmoset VGluT2 (GenBank accession number XM_002755104.3) & human VGluT2 (GenBank accession number NM_020346.1).

**How to cite this article**: Root, D. H. *et al*. Glutamate neurons are intermixed with midbrain dopamine neurons in nonhuman primates and humans. *Sci. Rep.* **6**, 30615; doi: 10.1038/srep30615 (2016).

## Figures and Tables

**Table 1:** Marmoset VTA neurons expressing VGluT2 mRNA or TH-immunoreactivity.

VTA Subdivision	Subject	VGluT2-only	VGluT2-TH	TH-only
VTAR	Turk	93.98% (n = 234)	3.61% (n = 9)	2.41% (n = 6)
	Dew	83.67% (n = 456)	7.16% (n = 39)	9.17% (n = 50)
	Wen	73.06% (n = 198)	7.38% (n = 20)	19.56% (n = 53)
	Mean ± SEM	83.57% ± 6.04%	6.05% ± 1.22%	10.38% ± 4.99%
RLi	Turk	58.95% (n = 313)	3.01% (n = 16)	38.04% (n = 202)
	Dew	41.06% (n = 216)	3.04% (n = 16)	55.89% (n = 294)
	Wen	56.2% (n = 272)	2.89% (n = 14)	40.91% (n = 198)
	Mean ± SEM	52.07% ± 5.56%	2.98% ± 0.05%	44.95% ± 5.54%
CLi	Turk	25.42% (n = 30)	1.69% (n = 2)	72.88% (n = 86)
	Dew	25.76% (n = 17)	1.52% (n = 1)	72.73% (n = 48)
	Wen	13.73% (n = 7)	1.96% (n = 1)	84.31% (n = 43)
	Mean ± SEM	21.64% ± 3.96%	1.72% ± 0.13%	76.64% ± 3.84%
VTAC	Turk	41.27% (n = 26)	1.59% (n = 1)	57.14% (n = 36)
	Dew	34.38% (n = 11)	9.38% (n = 3)	56.25% (n = 18)
	Wen	40.82% (n = 20)	4.08% (n = 2)	55.1% (n = 27)
	Mean ± SEM	38.82% ± 2.23%	5.01% ± 2.3%	56.16% ± 0.59%
IF	Turk	16.48% (n = 15)	1.1% (n = 1)	82.42% (n = 75)
	Dew	25% (n = 30)	5% (n = 6)	70% (n = 84)
	Wen	30.7% (n = 35)	0% (n = 0)	69.3% (n = 79)
	Mean ± SEM	24.06% ± 4.13%	2.03% ± 1.52%	73.91% ± 4.26%
PN	Turk	21.43% (n = 24)	0.89% (n = 1)	77.68% (n = 87)
	Dew	18.32% (n = 37)	3.96% (n = 8)	77.72% (n = 157)
	Wen	42.47% (n = 31)	1.37% (n = 1)	56.16% (n = 41)
	Mean ± SEM	27.4% ± 7.58%	2.07% ± 0.95%	70.52% ± 7.18%
PIF	Turk	19.44% (n = 7)	2.78% (n = 1)	77.78% (n = 28)
	Dew	20% (n = 29)	7.59% (n = 11)	72.41% (n = 105)
	Wen	19.05% (n = 4)	0% (n = 0)	80.95% (n = 17)
	Mean ± SEM	19.5% ± 0.28%	3.45% ± 2.22%	77.05% ± 2.49%
PBP	Turk	30.89% (n = 303)	11.42% (n = 112)	57.7% (n = 566)
	Dew	17.44% (n = 198)	23.17% (n = 263)	59.38% (n = 674)
	Wen	20.48% (n = 144)	19.49% (n = 137)	60.03% (n = 422)
	Mean ± SEM	22.94% ± 4.07%	18.03% ± 3.47%	59.04% ± 0.7%

Percent refers to the number of neurons expressing VGluT2 mRNA without TH-immunoreactivity (VGluT2-only neurons), neurons co-expressing VGluT2 mRNA and TH-immunoreactivity (VGluT2-TH neurons), or neurons expressing TH-immunoreactivity without VGluT2 mRNA (TH-only neurons) divided by the total number of labeled neurons identified in each subject and in each subdivision of the VTA. Numbers in parentheses refer to number of counted neurons of each phenotype.

**Table 2:** Marmoset SNC neurons expressing VGluT2 mRNA or TH-immunoreactivity.

SNC Subdivision	Subject	VGluT2-only	VGluT2-TH	TH-only
SNCd	Turk	3.04% (n = 32)	0.1% (n = 1)	96.86% (n = 1019)
	Dew	2.76% (n = 37)	0.07% (n = 1)	97.16% (n = 1302)
	Wen	1.7% (n = 21)	0.49% (n = 6)	97.81% (n = 1205)
	Mean ± SEM	2.5% ± 0.41%	0.22% ± 0.13%	97.28% ± 0.28%
SNCv	Turk	0% (n = 0)	0% (n = 0)	100% (n = 261)
	Dew	0% (n = 0)	0% (n = 0)	100% (n = 556)
	Wen	0% (n = 0)	0% (n = 0)	100% (n = 287)
	Mean ± SEM	0% ± 0%	0% ± 0%	100% ± 0%
SNCm	Turk	2.55% (n = 4)	0% (n = 0)	97.45% (n = 153)
	Dew	1.81% (n = 8)	0.23% (n = 1)	97.96% (n = 432)
	Wen	2.5% (n = 5)	0% (n = 0)	97.5% (n = 195)
	Mean ± SEM	2.29% ± 0.24%	0.08% ± 0.08%	97.64% ± 0.16%
SNCl	Turk	9.55% (n = 15)	0% (n = 0)	90.45% (n = 142)
	Dew	20.6% (n = 62)	0% (n = 0)	79.4% (n = 239)
	Wen	28.13% (n = 63)	0% (n = 0)	71.88% (n = 161)
	Mean ± SEM	19.43% ± 5.39%	0% ± 0%	80.57% ± 5.39%

Percent refers to the number of neurons expressing VGluT2 mRNA without TH-immunoreactivity (VGluT2-only neurons), neurons co-expressing VGluT2 mRNA and TH-immunoreactivity (VGluT2-TH neurons), or neurons expressing TH-immunoreactivity without VGluT2 mRNA (TH-only neurons) divided by the total number of labeled neurons identified in each subject and in each subdivision of the SNC. Numbers in parentheses refer to number of counted neurons of each phenotype.

**Table 3:** Human VTA neurons expressing VGluT2 mRNA or TH-immunoreactivity.

VTA Subdivision	Subject	VGluT2-only	VGluT2-TH	TH-only
RLi	Subject 1	49.48% (n = 383)	14.86% (n = 115)	35.66% (n = 276)
	Subject 2	34.6% (n = 228)	5.16% (n = 34)	60.24% (n = 397)
	Mean ± SEM	42.04% ± 7.44%	10.01% ± 4.85%	47.95% ± 12.29%
PBP	Subject 1	11.47% (n = 53)	27.92% (n = 129)	60.61% (n = 280)
	Subject 2	27.43% (n = 234)	6.8% (n = 58)	65.77% (n = 561)
	Mean ± SEM	19.45% ± 7.98%	17.36% ± 10.56%	63.19% ± 2.58%
VTA_SUB_	Subject 1	9.85% (n = 32)	9.54% (n = 31)	80.62% (n = 262)
	Subject 2	17.14% (n = 60)	10% (n = 35)	72.86% (n = 255)
	Mean ± SEM	13.49% ± 3.65%	9.77% ± 0.23%	76.74% ± 3.88%
PN	Subject 1	16.76% (n = 63)	2.66% (n = 10)	80.59% (n = 303)
	Subject 2	9.8% (n = 20)	0.49% (n = 1)	89.71% (n = 183)
	Mean ± SEM	13.28% ± 3.48%	1.57% ± 1.08%	85.15% ± 4.56%

Percent refers to the number of neurons expressing VGluT2 mRNA without TH-immunoreactivity (VGluT2-only neurons), neurons co-expressing VGluT2 mRNA and TH-immunoreactivity (VGluT2-TH neurons), or neurons expressing TH-immunoreactivity without VGluT2 mRNA (TH-only neurons) divided by the total number of labeled neurons identified in each subject and in each subdivision of the VTA. Numbers in parentheses refer to number of counted neurons of each phenotype.

**Table 4:** Human SNC neurons expressing VGluT2 mRNA or TH-immunoreactivity.

SNC Subdivision	Subject	VGluT2-only	VGluT2-TH	TH-only
SNCd	Subject 1	1.8% (n = 4)	2.7% (n = 6)	95.5% (n = 212)
	Subject 2	3.45% (n = 33)	2.61% (n = 25)	93.94% (n = 899)
	Mean ± SEM	2.63% ± 0.82%	2.66% ± 0.05%	94.72% ± 0.78%
SNCv	Subject 1	0% (n = 0)	0% (n = 0)	100% (n = 40)
	Subject 2	1.07% (n = 18)	3.2% (n = 54)	95.74% (n = 1617)
	Mean ± SEM	0.53% ± 0.53%	1.6% ± 1.6%	97.87% ± 2.13%
SNCm	Subject 1	1.78% (n = 6)	0.3% (n = 1)	97.92% (n = 330)
	Subject 2	1.19% (n = 9)	0.4% (n = 3)	98.42% (n = 747)
	Mean ± SEM	1.48% ± 0.3%	0.35% ± 0.05%	98.17% ± 0.25%
SNCl	Subject 1	19.05% (n = 12)	4.76% (n = 3)	76.19% (n = 48)
	Subject 2	7.73% (n = 28)	1.1% (n = 4)	91.16% (n = 330)
	Mean ± SEM	13.39% ± 5.66%	2.93% ± 1.83%	83.68% ± 7.48%

Percent refers to the number of neurons expressing VGluT2 mRNA without TH-immunoreactivity (VGluT2-only neurons), neurons co-expressing VGluT2 mRNA and TH-immunoreactivity (VGluT2-TH neurons), or neurons expressing TH-immunoreactivity without VGluT2 mRNA (TH-only neurons) divided by the total number of labeled neurons identified in each subject and in each subdivision of the SNC. Numbers in parentheses refer to number of counted neurons of each phenotype.
